# The mutation profile of JAK2 and CALR in Chinese Han patients with Philadelphia chromosome-negative myeloproliferative neoplasms

**DOI:** 10.1186/s13045-014-0048-6

**Published:** 2014-07-15

**Authors:** Zhiyuan Wu, Xinju Zhang, Xiao Xu, Yuming Chen, Tingting Hu, Zhihua Kang, Shibao Li, Hua Wang, Weiwei Liu, Xiaochao Ma, Ming Guan

**Affiliations:** 1 Central Laboratory, Huashan Hospital, Shanghai Medical College, Fudan University, Shanghai, People’s Republic of China; 2 Department of Laboratory Medicine, Huashan Hospital North, Shanghai Medical College, Fudan University, Shanghai, People’s Republic of China; 3 Department of Laboratory Medicine, Huashan Hospital, Shanghai Medical College, Fudan University, Shanghai, People’s Republic of China; 4 Department of Laboratory Medicine, Tenth People’s Hospital, Tongji University, Shanghai, People’s Republic of China; 5 Center for Pharmacogenetics, Department of Pharmaceutical Sciences, School of Pharmacy, University of Pittsburgh, Pittsburgh, Pennsylvania

## Abstract

Mutations in JAK2, MPL and CALR are highly relevant to the Philadelphia chromosome (Ph)-negative myeloproliferative neoplasms (MPNs). We performed high resolution melting analysis and Sanger sequencing together with T-A cloning to elucidate the unique mutation profile of these genes, in Chinese patients with MPNs. Peripheral blood DNA samples were obtained from 80 patients with polycythemia vera (PV), 80 patients with essential thrombocytosis (ET) and 50 patients with primary myelofibrosis (PMF). Ten PV patients were identified with diverse JAK2 exon 12 mutations. Five novel JAK2 Exon 12 mutation patterns (M532V/E543G, N533D, M535I/H538Y/K549I, E543G and D544N) were described. JAK2 V617F was detected in 140 samples (66 PV, 45 ET and 29 PMF). JAK2 Exon 12 mutations were prevalent (13%) and variable in the Chinese patients. Compared with PV patients with JAK2 V617F mutations, PV patients with JAK2 exon 12 mutations had an earlier median onset of disease (P = 0.0013). MPL W515L/K mutations were discerned in 4 ET and 3 PMF patients. Two kinds of CALR mutation, c. 1179_1230del and c. 1234_1235insTTGTC were detected in 20 ET and 16 PMF patients. A novel CALR mutation pattern (c. 1173_1223del/c. 1179_1230del) was identified in 2 PMF samples. In addition, 17 scattered point mutations in CALR c.1153 to c.1255 were also detected in 13 cases with CALR frame-shifting variations and 2 cases without CALR frame-shifting variations. Female patients showed a predisposition to CALR mutations (P = 0.0035). Chinese Ph-negative MPN patients have a unique mutation landscape in the common molecular markers of MPN diagnosis. Validation of the molecular diagnostic pipeline should be emphasized since there is a considerable ethnical diversity in the molecular profiles of Ph-negative MPNs.

## Introduction

Myeloproliferative neoplasms (MPNs), the malignant conditions characterized by myeloid cell excessive proliferation, were first proposed by the American hematologist Dr. William Dameshek in 1951 [[1]]. Apart from chronic myelogenous leukemia (CML) characterized by the Philadelphia chromosome (Ph) [[2]], the classical Ph-negative MPNs comprise polycythemia vera (PV), essential thrombocytosis (ET) and primary myelofibrosis (PMF), according to the 2008 World Health Organization (WHO) classification criteria [[3]].

Routine diagnostic tests for MPNs include red cell mass determination, bone marrow aspirate and trephine biopsy, arterial oxygen saturation and carboxyhaemoglobin level, neutrophil akaline phosphatase level, vitamin B12 and serum urate [[4]]. Although these tests strongly support the diagnosis of MPNs, the disease could still not be well discriminated from the reactive hyperplasia. Moreover, misdiagnosis could occur when the symptoms of disease are atypical.

In 2005, somatic mutation in JAK2 exon 14 (JAK2 V617F) was first reported to occur in over 95% of PV and approximately 50% of ET and PMF patients, resulting in the auto-activation of this tyrosine kinase and enhanced signaling of the down-stream JAK-STAT pathway [[5],[6]]. Soon after this remarkable discovery, mutations in JAK2 exon 12 were reported to prevail in JAK2 V617F-negative MPNs patients, specifically those with PV in most cases [[7]]. Apart from the mutations in JAK2 gene, genetic alteration in MPL, mainly manifested as MPL W515L and W515K variation in the 10th exon, also plays an important role in the pathogenesis of ET and PMF [[8],[9]]. With a view to promote the molecular diagnosis of MPNs, these mutations were consequently integrated into the WHO diagnostic criteria in 2008 [[10]].

Recently, another milestone in the molecular diagnosis of MPNs, somatic mutations in the CALR gene, was reported [[11],[12]]. In terms of frame shifting insertion and deletion, these JAK2- and MPL-exclusive mutations were found to present in 30% to 40% of ET and PMF patients and were considered highly likely to be integrated into the next version of MPN diagnostic criteria [[13],[14]].

Although the molecular variation in Ph-negative MPNs patients is increasingly a concern for both scientific researchers and clinical professionals, the panoramagram of major genetic alternations, which could be highly variable between the different ethnicities [[15]], has not been depicted in Chinese Han patients with MPNs.

In this study, we assessed the major mutations in the JAK2, MPL and CALR genes in 210 Chinese Han MPNs patients, employing high resolution melting curve analysis (HRMA) for preliminary screening and Sanger sequencing for mutation validation, in order to unveil the MPN-specific mutations in the Chinese Han population.

## Materials and methods

### Patients

Peripheral blood samples were obtained from 80 PV, 80 ET and 50 PMF patients in the Department of Hematology in Huashan Hospital and Department of Hematology of Shanghai Tenth People’s Hospital. All these Ph-negative patients were diagnosed according to the *WHO Classification of Tumours of Haematopoietic and Lymphoid Tissues (2008) *[[3]]. Written informed consent was received from all the participants. DNA was extracted from the blood samples collected in ethylenediaminetetraacetic acid anticoagulant with QiaAmp DNA Blood Mini kit, and diluted with ddH_2_O to a final concentration of 15–25 ng/μl. In compliance with Helsinki Declaration of 1975 as revised in 1996, this study was approved by the Institutional Review Board of Huashan Hospital. The patients’ clinical features, including age, gender and hematological test results were illustrated in Table 1.

**Table 1:** Clinical and laboratory features of 210 patients with myeloproliferative neoplasms, stratified by the clinical diagnosis of polycythemia vera (PV), essential thrombocytosis (ET) and primary myelofibrosis (PMF)

**Variables**	**PV (n = 80)**	**ET (n = 80)**	**PMF (n = 50)**	**All patients (n = 210)**
Age in years; median (range)	61 (20–89)	59 (19–94)	60 (36–89)	60 (19–94)
Age > 60 years; n (%)	51 (63%)	45 (56%)	31 (62%)	127 (60%)
Leukocytes, × 10^9^/l; median (range)	10.94 (1–42.41)	9.94 (2.83–22.71)	10.38 (0.69–92.67)	10.43 (0.69–92.67)
Erythrocytes, × 10^12^/l; median (range)	6.36 (2.22–9.93)	4.53 (1.51–6.84)	4.11 (1.97–9.36)	5.15 (1.51–9.93)
Hemoglobin, g/dl; median (range)	186.25 (73–245)	132.94 (48–182)	116.95 (44–278)	150.22 (44–278)
Platelets, × 10^9^/l; median (range)	292.61 (56–715)	774.89 (328–2887)	416.30 (16–2890)	509.47 (16–2890)

### Mutation screening of JAK2 Exon 12 with high resolution melting analysis

High-resolution melting analysis (HRMA) was used to screen for genetic alternations in JAK2 Exon 12. The whole Exon 12 was amplified by PCR with the mutation screen primers (sequences described in Table 2), amplifying a 127 bp small amplicon in order to achieve a high mutation detecting sensitivity [[16]]. Each PCR reaction was performed in a 50 μl of reaction volume. The master mix contained: 2× Premix ExTaq Hotstart (TaKaRa BIO, Shiga, Japan), 0.25 μM E12HRM-F primer, 0.25 μM E12HRM-R primer, 1.5 μM SYTO-9 DNA dye (Invitrogen, Carlsbad, CA) and 15-25 ng DNA template. The mix was subjected to an ABI 9700 thermal cycler (Applied Biosystems, Foster City, CA) for the gene amplification. The PCR protocol included an initial denaturation of 95°C for 10 min, followed by 35 cycles of 95°C for 30 sec, 58°C for 30 sec, and 72°C for 30 sec. The products were then brought into the HRMA assay on a Rotor-Gene Q HRMA platform (Qiagen, Germany). During HRMA, these amplicons were heated to 98°C for 2 min and then cooled to 40°C in order to form the abundant heteroduplex; then they were melted at a ramping rate of 0.1°C/s from 65°C to 85°C. The melting fluorescent data were collected using Rotor-Gene Q 1.7 software. For each assay, 2 peripheral blood DNA samples obtained from the healthy people were also amplified and melted as the wild-type control. Any sample identified with a melting curve not fitting that of the wild-type control was considered as a mutation suspect.

**Table 2:** Primers for JAK2 Exon 12, JAK2 V617F and CALR mutation screening and sequencing

	**Primer**	**Sequence (5’-3’)**	**Amplicon length (bp)**
JAK2 exon 12 mutation screening	E12HRM-F	AATGGTGTTTCTGATGTACC	127
	E12HRM-R	AGACAGTAATGAGTATCTAATGAC	
JAK2 exon 12 sequencing	E12SEQ-F	CTCCTCTTTGGAGCAATTCA	496
	E12SEQ-R	GAGAACTTGGGAGTTGCGATA	
JAK2 V617F	V617F-F	AGCTTTCTCACAAGCATTTGG	150
	V617F-R	TGACACCTAGCTGTGATCCTG	
	V617F-P	AAATTATGGAGTATGTTTCTGTGGAGACGAGA	
CALR exon sequencing	CALRExon1-F	CGGGTGGGTATAAAAGTG	348
	CALRExon1-R	GGGACGCAGAAGAGAAAT	
	CALRExon2-F	GTTGGAATGGGGAGTGTC	337
	CALRExon2-R	CTTCCTCCACCTGTCCTC	
	CALRExon3-F	CGGTGACGAGGAGAAAGATA	493
	CALRExon3-R	TAAGAAAGTCAATGGGGTCT	
	CALRExon4.5-F	GTTTCTCTTCTCAGCCTTG	678
	CALRExon4.5-R	TCTCTTCATCCCAGTCCTC	
	CALRExon6.7-R	ATCCCACAGACTCCAAGC	611
	CALRExon6.7-R	GGACTTCACAGGGACAGAC	
	CALRExon8.9-F	CGGTGTTCCTTGTCTTCTC	584
	CALRExon8.9-R	ACAGAGACATTATTTGGCG	

### Genotyping of JAK2 Exon 12 mutations

Sanger sequencing was further applied to validate the suspected JAK2 Exon 12 mutations. The sequencing primers flanked the intron 11, exon 12 and intron 12 of the JAK2 gene (sequences described in Table 2). Gene amplification was carried out in a 20 μl PCR mix, which contained 2× Premix ExTaq Hotstart, 0.25 μM E12SEQ-F primer, 0.25 μM E12SEQ-R primer and 15-25 ng DNA template with the thermal cycling procedure described in the *Mutation Screening of JAK2 Exon 12 with High Resolution Melting* section. After the amplification, all the products were separated by agarose electrophoresis and delivered to the Technical Support Department of BGI genomics institution (Shanghai, China) for the subsequent bi-directional sequencing procedure on an ABI 3730 XL genetic analyzer (Applied Biosystems).

Since the JAK2 exon 12 mutation enjoys a predisposition to rare allele load [[17]], T-A cloning was performed to isolate the pure mutant subclones for accurate mutation genotyping before the final conformation. For each JAK2 exon 12 mutation candidate, the PCR amplicon was ligated to the PMD19-T simple vector (Takara), and then transformed into DH5α competent E. coli cells. The bacteria were proliferated in Luria-Bertani broth and spread onto the IPTG-Xgel (Invitrogen) coated ampicillin-LB araga dishes for blue/white selection, and plasmids were extracted from the enriched white isolations with Plasmid Mini Kit (Qiagen). For each sample, 20 bacteria clones were isolated for the subsequent DNA sequencing.

### Detection of JAK2 V617F mutation with unlabeled probe melting assay

As we previously described, the unlabeled probe melting technology is a robust tool for identifying the JAK V617F mutation [[18]]. This unlabeled probe HRMA was again performed on the Rotor-Gene Q platform to identify the JAK2 V617F mutation in the peripheral DNA of 210 MPNs patients.

### Mutation scanning of MPL W515L and W515K mutations with Taqman qPCR

All 130 ET and PMF samples were subjected to a Taqman probe based qPCR assay [[19]] to detect the MPL W515L and MPL W515K mutations. The qPCR procedure was carried out with the Quantitect probe qPCR kit (Qiagen) on the Rotor-Gene Q instrument according to the manufacturer’s instructions, and the data analysis was finished with the Rotor-Gene Q 1.7 software.

### Sanger sequencing of CALR gene

Five different DNA poolings were prepared for the sequencing of all 9 exons in CALR. Each pooling comprised 3 equivalent portions of peripheral blood DNA from diverse JAK2/MPL mutation-free ET or PMF patients, to ensure the final sequencing data embodied the genetic information in CALR exons from 15 patients. These poolings were amplified with the sequencing primers (sequences listed in Table 2) and gene amplification procedure described in the *Genotyping of JAK2 Exon 12 Mutations* section. Agarose electrophoresis purified amplicons were then subjected to bi-directional Sanger sequencing. Moreover, all 210 peripheral blood DNA samples were amplified with sequencing primer targeting the CALR Exon 9 (CALRExon8.9-F and CALRExon8.9-R). Purified amplicons were also bi-directionally sequenced. All DNA sequencing experiments were carried out on an ABI XL3730 genetic analyzer in the BGI Shanghai laboratory. For each mutation-susceptive sample, T-A cloning was also used to confirm the mutation with pure mutant subclones.

### Mutation calling in JAK2 Exon 12 and CALR

All the sequencing results were integrated into the Mutation Surveyor V4.0.6 software (SoftGenetics, State College, PA) and then aligned with National Center for Biotechnology Information (NCBI) reference gene contigs (NM_004972.3 for JAK2 and NM_004343.3 for CALR), respectively. To eliminate the false positives from regions of low data quality, we set the mutation trimming score to 20 for the final mutation calling.

### Statistical analysis

The clinical parameters were statistically analyzed with Mann–Whitney U test (for measurement data) or Fisher’s exact test (for enumeration data).

## Results

### Mutation screening of JAK2 Exon 12 by HRMA and genotyping

**Figure 1:**
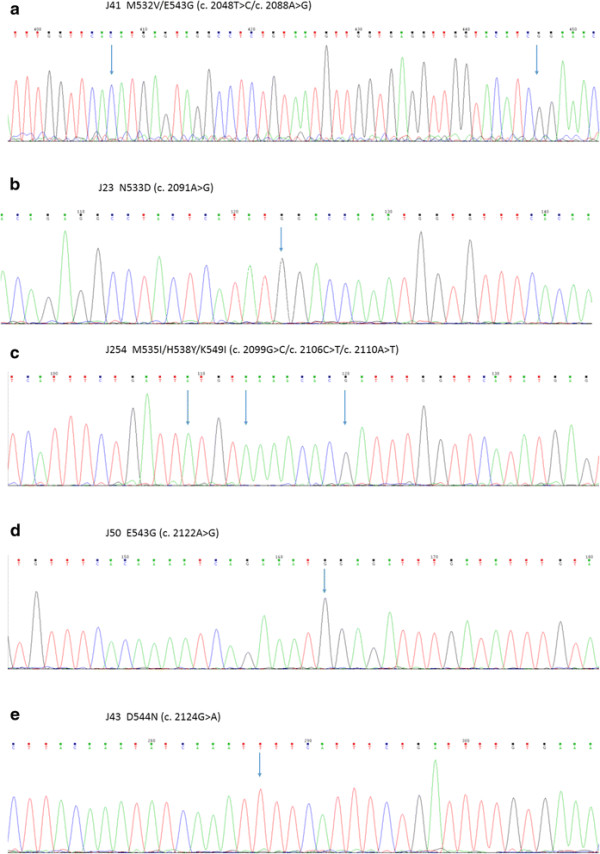
**Five novel mutation profiles in JAK2 exon 12.** Arrows indicate the mutation site. **a)** M532V/E543G (c. 2048 T > C/c. 2088A > G). **b)** N533D (c. 2091A > G). **c)** M535I/H538Y/K549I (c. 2099G > C/c. 2106C > T/c. 2110A > T). **d)** E543G (c. 2122A > G). **e)** D544N (c. 2124G > A). **a)**, **c)**, **e)**: with reverse sequencing primer. **b)**, **d)**: with forward sequencing primer.

The HRMA method discerned 10 JAK2 Exon 12 mutation positive samples from 200 MPN samples with wild-type Exon 12. All these mutation-susceptive samples came from patients diagnosed with PV or showed a distinctive erythremia-related feature. The mutation genotyping and conformation were carried out with further Sanger sequencing and T-A cloning. Among these 10 samples, we identified five novel genetic alternation patterns, M532V/E543G (c. 2048 T > C/c. 2088A > G), N533D (c. 2091A > G), M535I/H538Y/K549I (c. 2099G > C/c. 2106C > T/c. 2110A > T), E543G (c. 2122A > G) and D544N (c. 2124G > A) (Figure 1), besides the previously reported mutation models such as F537L (c. 2103 T > C) [http://www.cancer.sanger.ac.uk/cosmic/mutation/overview?id=1462560], F537-I546dup10/F547L [[20]], K539L, N542-E543del and H538K539delinsL [[7]]. The clinical parameters and the JAK2 Exon 12 mutation profiles of each patient are listed in Table 3.

**Table 3:** Clinical features of 10 myeloproliferative neoplasm patients with JAK2 Exon 12 mutations

**Patient no.**	**Clinical diagnosis**	**Age**	**Gender**	**JAK2 Exon 12 mutation**	**WBC (10E9/L)**	**RBC (10E12/L)**	**Hb (g/L)**	**PLT (10E9/L)**
J2	PV	44	Male	F537-I546dup10/F547L	14.25	3.94	161	438
J23	PV	60	Male	N533D	9.93	4.45	153	136
J27	PV with splenomegaly	44	Female	F537L	N/A*			
J32	PV	43	Male	K539L	7.57	5.83	182	159
J41	PV	35	Male	M532V/E543G	8.33	5.72	180	192
J43	PV with recurrent brain stem hemorrhage	47	Male	D544N	9.11	3.86	112	494
J50	PV	44	Male	E543G	14.46	5.1	166	257
J71	PV	41	Female	N542-E543del	6.46	4.14	143	293
J207	PV	43	Female	H538K539delinsL	5.85	5.81	178	85
J254	PV	23	Female	M535I/H538Y/K539I	7.08	7.46	216	151

*Complete blood count was not available as the patient was in an impaired hematopoiesis condition

### JAK2 V617F Mutation Screening with Unlabeled Probe Melting Assay

The unlabeled probe melting JAK2 V617F detecting system indicated there were 66 (82%) PV, 45 (56%) ET and 29 (58%) PMF cases in the MPN patient cohort. No concurrent JAK2 Exon 12/Exon14 (V617F) mutation was identified in any of these samples.

Compared with those PV patients afflicted with JAK2 exon 12 mutations, V617F-positive PV patients showed a delayed median disease onset (P = 0.0013). Among all 140 JAK2 V617F-positive MPNs patients, 124 (88%) were over 60 years old. However, there was no statistical difference according to gender and complete blood cell count (CBC) in the PV patients.

### Identification of MPL W515L and W515K mutations with Taqman Probe qPCR

The qPCR method identified 3 MPL W515L mutant and 1 MPL W515K mutant ET samples, and 2 W515L and 1 W515K mutant PMF cases. All these 7 samples were with the wild-type JAK2 exon 12 and exon 14.

### CALR mutations in the MPN patients

**Figure 2:**
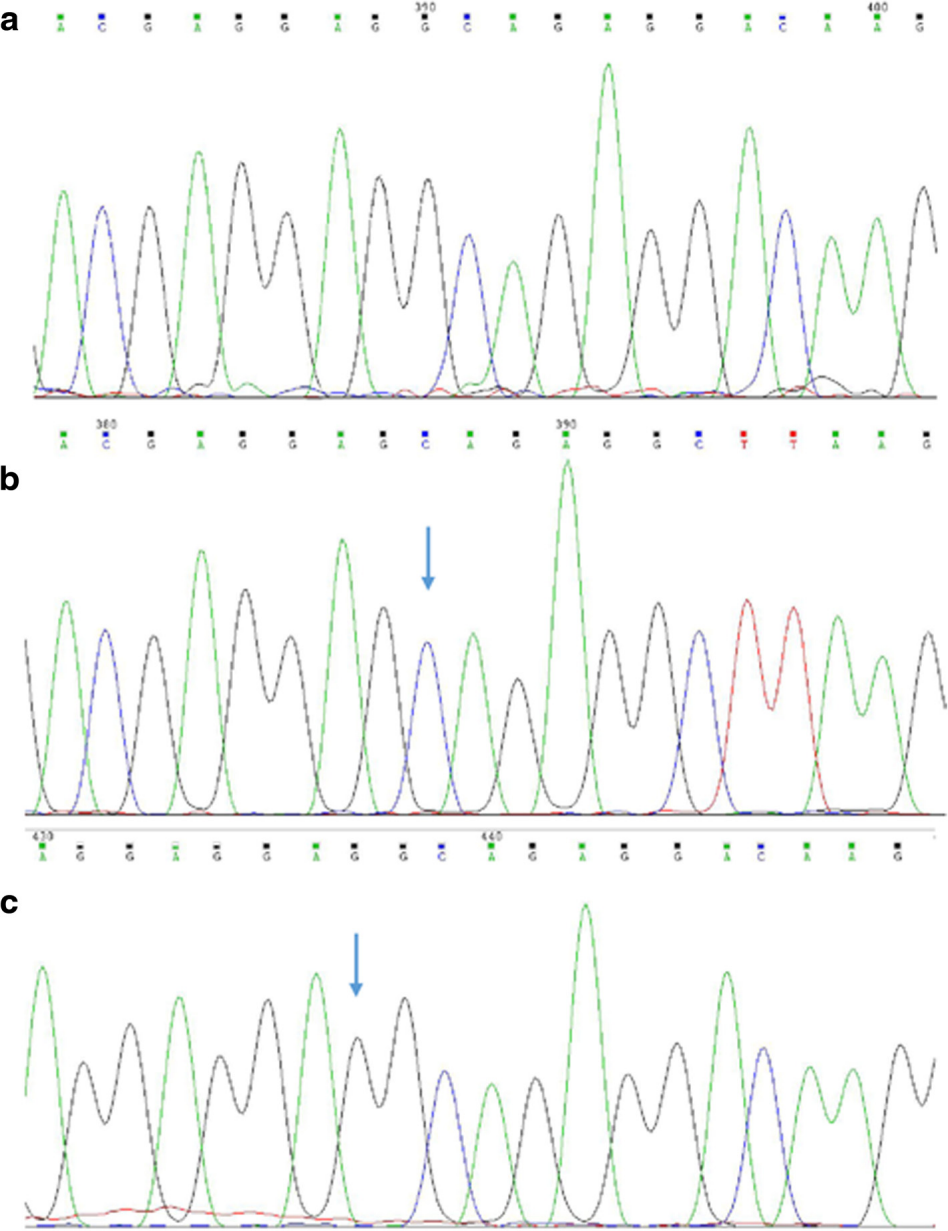
**CALR c. 1173_1223del mutation.** The 51 bp deletion was detected by Sanger sequencing using forward sequencing primer. **a)** CALR c. 1173_1223del sequence. **b)** wild-type sequence. Arrow indicates c. 1173 truncating site. **c)** wild-type sequence. Arrow indicates c. 1223 truncating site.

In the 5 poolings of MPN patients’ DNA, neither novel frame shifting nor recurrent point mutation was detected within all the 9 CALR exons. The mutations in CALR exon 9 were further screened and confirmed by the bi-directional Sanger sequencing and T-A cloning in all 210 samples. A total of 36 patients were found to harbor the CALR mutations. Twenty of these patients were diagnosed with ET, while the remainder were characterized with the PMF diagnosis. The previously reported 52 bp deletion (c. 1179_1230del) and 5 bp insertion (c. 1234_1235insTTGTC) were detected in 17 and 19 patients, respectively. Moreover, we also identified one new kind of CALR genetic variation, c. 1173_1223del in the PCR amplicon subclones, which was derived from two independent PMF samples with the c. 1179_1230del mutation (Figure 2).

Twenty seven (75%) of the 36 patients identified with CALR insertion or deletion were female, suggesting the female patients showed a vulnerability to the CALR mutation (P = 0.0035). No further statistical difference was observed between the clinical parameters of CALR mutation-positive cases and the CALR mutation-free ones.

**Figure 3:**
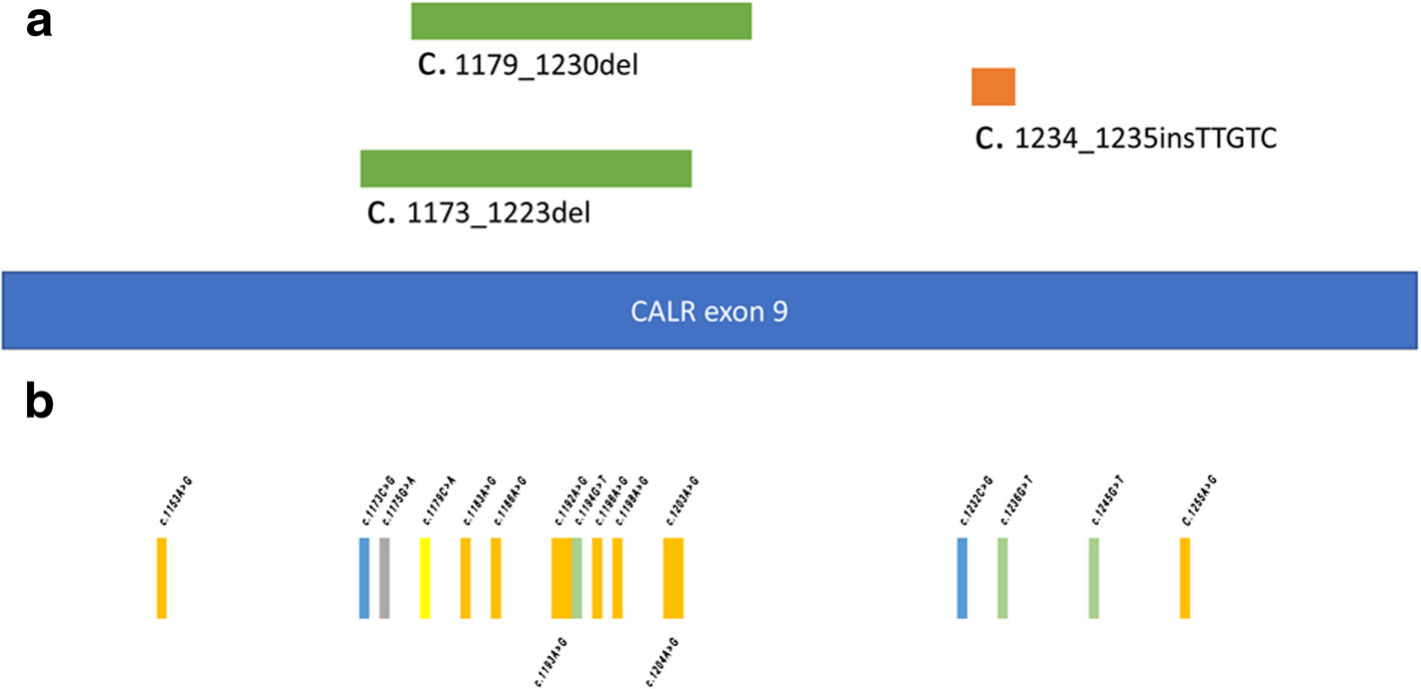
**Mutations in CALR exon 9. a)** Three types of CALR exon 9 frame-shifting mutations **(c**. 1173_1223del, c. 1179_1230del, 1234_1235insTTGTC). **b)** 17 scattered point mutations in c.1153 to c.1255.

It is worth noting that we found 17 non-recurrent point mutations accumulated in the CALR Exon 9 in 1 ET and 2 PMF cases free of CALR exon 9 insertion or deletion and in 6 ET and 6 PMF cases with the frame shifting mutations (Figure 3 and Additional file 1: Figure S1). The detailed mutation profile of each patient is listed in Table 4.

**Table 4:** CALR exon 9 point mutation profile in 15 MPN patients

**Patient no.**	**Clinical diagnosis**	**CALR exon 9 frame-shifting mutations**	**CALR exon 9 point mutations**
J31	PMF	c. 1173_1223del/c. 1179_1230del	c. 1255A > G
J91	PMF	c. 1234_1235insTTGTC	c. 1232C > G
J94	PMF	c. 1179_1230del	c. 1173C > G
J101	PMF	c. 1234_1235insTTGTC	c. 1203A > G
J167	PMF	Undetected	c. 1175G > A
J172	PMF	c. 1234_1235insTTGTC	c. 1153A > G
J183	ET	c. 1179_1230del	c. 1196A > G/c. 1204A > G
J211	PMF	Undetected	c.1186A > G
J233	ET	c. 1234_1235insTTGTC	c. 1236G > T
J243	PMF	c. 1173_1223del/c. 1179_1230del	c. 1198A > G
J259	ET	c. 1179_1230del	c. 1179C > A/c. 1193A > G
J265	ET	c. 1234_1235insTTGTC	c. 1194G > T
J296	ET	Undetected	c. 1183A > G
J307	ET	c. 1179_1230del	c. 1245G > T
J312	ET	c. 1234_1235insTTGTC	c. 1192A > G

Compared with those ET and PMF patients with JAK2 V617F or MPL mutations, the CALR mutant patients showed a lower white blood cell count (P = 0.0061), elevated platelet (P = 0.0168) and younger age (P = 0.0002).

## Discussion

Since the first description of myeloproliferative diseases by Dr. William Dameshek in 1951 [[1]], there has been a consecutive progression in the understanding of these disease conditions characterized by abnormal bone marrow hyperplasia. Apart from the characterization of the Philadelphia chromosome in the CML, the discovery of the JAK2 V617F mutation in 2005 [[5],[6]] is the most thrilling development in the molecular diagnosis of Ph-negative MPNs. The subsequently reported somatic mutation in JAK2 exon 12 [[7]], though much less prevalent in the patients, is considered as another robust molecular marker for Ph-negative MPNs, especially for PV patients.

Apart from these established pathological markers in JAK2, the genetic mutations in MPL, especially the W515L and W515K substitution in exon 10 could also play a role in the diagnosis of ET and PMF [[21],[22]].

Recently, a total of 36 types of frame-shifting insertions and deletions were detected in the CALR gene, which encodes a Ca^2+^ binding protein in endoplasmic reticulum called calreticulin. These mutations are located in the 9th exon of the gene, leading to the imperilment of the protein’s C-terminal structure, and were reported to have a incidence of over 60% to 80% in JAK2 and MPL mutation-negative ET and PMF patients [[11],[12]].

In this study, we used the preliminary HRMA and Sanger sequencing method, together with the T-A cloning strategy to reveal the unique genetic variation background of JAK2, MPL and CALR in Chinese Ph-negative MPN patients.

JAK2 V617F is the most prevalent genetic alternation in MPN patients, which could be detected in approximately 95% of patients with PV [[23],[24]]. However, we noticed the Chinese PV patients have a relatively low mutation frequency in the JAK2 V617F mutation (82%), while the mutations in JAK2 exon 12 were much more pervasive (13%) than in the Westerns [[25],[26]], and other East Asians [[27],[28]]. This observation is compatible with the previous small-scale investigation from Taiwan [[29]]. Meanwhile, all the 10 JAK2 Exon 12-mutant patients showed a unique genetic variation pattern, half of which has not been previously reported, indicating the mutation patterns in the JAK2 Exon 12 were highly diverse in the Chinese PV patients.

In our patient cohort, the JAK2 exon 12 mutations were inclined to affect young and middle-aged patients, while the V617F mutation was predominantly found in patients over age 60, which was consistent with a previous report [[30]]. In addition, the high prevalence of exon12 highlights its detection value in Chinese PV patients.

Compared with the JAK2 mutations, genetic abnormalities in MPL exon 10, namely the MPL W515L and W515K mutations, are rare but specific markers for ET and PMF patients. In the Chinese MPN patients, these two types of MPL mutations could be found in approximately 5% of ET and 6% of PMF patients.

For the CALR gene, we identified a novel type of exon 9 long-fragment deletion, c. 1173_1223del, which was isolated in the subclones from patients with the previously reported c. 1179_1230del deletion in exon 9. Meanwhile, the overwhelming majority (94%) of the CALR mutation pattern still lies in the c. 1179_1230del and c. 1234_1235insTTGTC. Our data also indicated that female patients were predisposed to the CALR insertion/deletion. Moreover, we also found a number of point mutations accumulated in c.1153 to c.1255 of the CALR gene, in both the patients with and without the exon 9 insertion/deletion. This phenomenon implied that these scattered point mutations could be the predecessors of the frame-shifting alternation and indicated that the genetic alternations in CALR exon 9 could be an acquired clonal evolution event [[31],[32]]. Although we extracted the data from a moderately-sized patient cohort, this is the first time the landscape of CALR mutations in Chinese MPN patients was revealed.

As reported in the previous literature, a number of gene loci are frequently affected by complicated mutations in MPN patients, such as those in JAK2, CALR, TET2, DNMT3A, and ASXL1 [[33]–[36]]. However, the mechanism behind the pathogenesis and development of these MPNs relevant to genetic imperilment is still unexplored. Given the high variability of Ph-negative MPN relevant gene mutations, we hypothesize that the Ph-negative MPN-related gene variation could be a sequel to the structural instability of nucleotide sequence, and the DNA repair pathway impairment may play a role in the early pathogenesis of MPNs [[37]].

**Figure 4:**
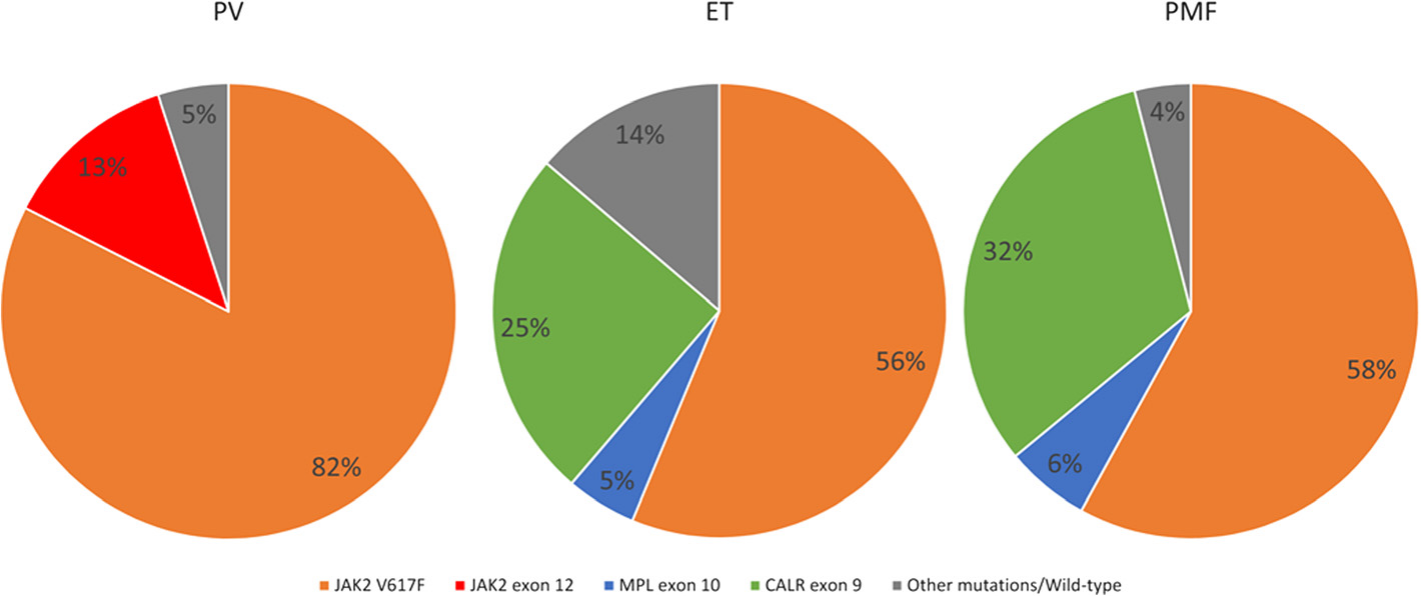
Variation frequency of JAK2 exon 12, JAK2 V617F, MPL W515L/K and CALR exon 9 frame-shifting mutations.

Mutations in JAK2 exon 12, exon 14, MPL exon 10 and CALR exon 9 were presented in each individual exclusively. Patients with genetic alternations in these three genes accounted for up to 90% of all the recruited patients (Figure 4). The highlighted prevalence of these mutations provides us with a multi-gene scanning strategy that has favorable sensitivity for the diagnosis of the Ph-negative MPNs.

JAK2 V617F and CALR mutations lead to disparate clinical features in the ET and PMF patients. Several previous publications reported the the higher platelet count and lower leukocyte and hemoglobin values in CALR mutant ET patients compared with those with JAK2 V617F or MPL mutations [[38]–[40]]. Our findings confirmed part of those findings (elevated platelet, decreased hemoglobin and earlier disease onset) in the Chinese Han population. However, the different gender inclination in CALR mutation prevalence may be due to the ethnic dissimilarity and sample size.

Despite the progress in the development of MPN molecular markers, the diagnosis of individual patients with MPNs has been a complicated issue demanding intensive collaboration between the clinic and laboratory [[41]]. Our results shed light on a combined molecular diagnostic model, which utilizes the most prevalent DNA variation signatures in three MPN pathogenesis related genes. Moreover, we also suggest that the validation of the molecular diagnostic pipeline should be heeded since there is a considerable ethnical diversity in the molecular profiles of Ph-negative MPNs.

## Abbreviations

Ph: Philadelphia chromosome

MPNs: Myeloproliferative neoplasms

CML: Chronic myelogenous leukemia

PV: Polycythemia vera

ET: Essential thrombocytosis

PMF: Primary myelofibrosis

HRMA: High resolution melting curve analysis

CBC: Complete blood cell count

## Competing interests

The authors declare that they have no competing interests.

## Authors’ contributions

WZ, ZX, and XX carried out the molecular genetic studies and participated in the sequence alignment. WZ drafted the manuscript. CY and HT performed the statistical analysis. KZ, LS, WH helped to draft the manuscript. WZ, LW, MX and GM participated in the design of the study. All the authors read and approved the final manuscript.

## Additional file

## Supplementary Material

Additional file 1: Figure S1. Seventeen Scattered Point Mutations in CALR Exon 9. Click here for file

## References

[B1] Dameshek W Editorial: some speculations on the myeloproliferative syndromes Blood 1951 6 372 375 14820991

[B2] Nowell PC A minute chromosome in human chronic granulocytic leukemia Science 1960 132 1497

[B3] Swerdllow S Campo E Harris NL Who Classification Of Tumours Of Haematopoietic And Lymphoid Tissues 2008 IARC Press, France

[B4] Lewis SM Bain BJ Bates I Dacie JV Dacie And Lewis Practical Haematology 2006 Elsevier Health Sciences, Philadelphia

[B5] James C Ugo V Le Couédic J-P Staerk J Delhommeau F Lacout C Garçon L Raslova H Berger R Bennaceur-Griscelli A A unique clonal JAK2 mutation leading to constitutive signalling causes polycythaemia vera Nature 2005 434 1144 1148 10.1038/nature03546 15793561

[B6] Baxter EJ Scott LM Campbell PJ East C Fourouclas N Swanton S Vassiliou GS Bench AJ Boyd EM Curtin N Acquired mutation of the tyrosine kinase JAK2 in human myeloproliferative disorders Lancet 2005 365 1054 1061 10.1016/S0140-6736(05)71142-9 15781101

[B7] Scott LM Tong W Levine RL Scott MA Beer PA Stratton MR Futreal PA Erber WN McMullin MF Harrison CN JAK2 exon 12 mutations in polycythemia vera and idiopathic erythrocytosis N Engl J Med 2007 356 459 468 10.1056/NEJMoa065202 17267906 PMC2873834

[B8] Pikman Y Lee BH Mercher T McDowell E Ebert BL Gozo M Cuker A Wernig G Moore S Galinsky I MPLW515L is a novel somatic activating mutation in myelofibrosis with myeloid metaplasia PLoS Med 2006 3 e270 10.1371/journal.pmed.0030270 16834459 PMC1502153

[B9] Pardanani AD Levine RL Lasho T Pikman Y Mesa RA Wadleigh M Steensma DP Elliott MA Wolanskyj AP Hogan WJ MPL515 mutations in myeloproliferative and other myeloid disorders: a study of 1182 patients Blood 2006 108 3472 3476 10.1182/blood-2006-04-018879 16868251

[B10] Tefferi A Vardiman J Classification and diagnosis of myeloproliferative neoplasms: the 2008 World Health Organization criteria and point-of-care diagnostic algorithms Leukemia 2008 22 14 22 10.1038/sj.leu.2404955 17882280

[B11] Klampfl T Gisslinger H Harutyunyan AS Nivarthi H Rumi E Milosevic JD Them NC Berg T Gisslinger B Pietra D Somatic mutations of calreticulin in myeloproliferative neoplasms N Engl J Med 2013 369 2379 2390 10.1056/NEJMoa1311347 24325356

[B12] Nangalia J Massie CE Baxter EJ Nice FL Gundem G Wedge DC Avezov E Li J Kollmann K Kent DG Somatic CALR mutations in myeloproliferative neoplasms with nonmutated JAK2 N Engl J Med 2013 369 2391 2405 10.1056/NEJMoa1312542 24325359 PMC3966280

[B13] Tefferi A Thiele J Vannucchi A Barbui T An overview on CALR and CSF3R mutations and a proposal for revision of WHO diagnostic criteria for myeloproliferative neoplasms Leukemia 2014 ᅟ ᅟ [Epub ahead of print] 10.1038/leu.2014.35 24441292

[B14] Tefferi A Pardanani A Genetics: CALR mutations and a new diagnostic algorithm for MPN Nat Rev Clin Oncol 2014 11 125 126 10.1038/nrclinonc.2014.16 24514146

[B15] Levine RL Wadleigh M Cools J Ebert BL Wernig G Huntly BJ Boggon TJ Wlodarska I Clark JJ Moore S Activating mutation in the tyrosine kinase JAK2 in polycythemia vera, essential thrombocythemia, and myeloid metaplasia with myelofibrosis Cancer Cell 2005 7 387 397 10.1016/j.ccr.2005.03.023 15837627

[B16] Jones AV Cross NC White HE Green AR Scott LM Rapid identification of JAK2 exon 12 mutations using high resolution melting analysis Haematologica 2008 93 1560 1564 10.3324/haematol.12883 18698085

[B17] Theocharides A Passweg JR Medinger M Looser R Li S Hao-Shen H Buser AS Gratwohl A Tichelli A Skoda RC The allele burden of JAK2 mutations remains stable over several years in patients with myeloproliferative disorders Haematologica 2008 93 1890 1893 10.3324/haematol.13074 18790796

[B18] Wu Z Yuan H Zhang X Liu W Xu J Zhang W Guan M Development and inter-laboratory validation of unlabeled probe melting curve analysis for detection of JAK2 V617F mutation in polycythemia vera PLoS One 2011 6 e26534 10.1371/journal.pone.0026534 22028900 PMC3197667

[B19] Pancrazzi A Guglielmelli P Ponziani V Bergamaschi G Bosi A Barosi G Vannucchi AM A sensitive detection method for MPL W515L or MPL W515K mutation in chronic myeloproliferative disorders with locked nucleic acid-modified probes and real-time polymerase chain reaction J Mol Diagn 2008 10 435 441 10.2353/jmoldx.2008.080015 18669880 PMC2518738

[B20] Pietra D Li S Brisci A Passamonti F Rumi E Theocharides A Ferrari M Gisslinger H Kralovics R Cremonesi L Somatic mutations of JAK2 exon 12 in patients with JAK2 (V617F)-negative myeloproliferative disorders Blood 2008 111 1686 1689 10.1182/blood-2007-07-101576 17984312

[B21] Vannucchi AM Antonioli E Guglielmelli P Pancrazzi A Guerini V Barosi G Ruggeri M Specchia G Lo-Coco F Delaini F Characteristics and clinical correlates of MPL 515W> L/K mutation in essential thrombocythemia Blood 2008 112 844 847 10.1182/blood-2008-01-135897 18519816

[B22] Beer PA Campbell PJ Scott LM Bench AJ Erber WN Bareford D Wilkins BS Reilly JT Hasselbalch HC Bowman R MPL mutations in myeloproliferative disorders: analysis of the PT-1 cohort Blood 2008 112 141 149 10.1182/blood-2008-01-131664 18451306

[B23] Lippert E Boissinot M Kralovics R Girodon F Dobo I Praloran V Boiret-Dupré N Skoda RC Hermouet S The JAK2-V617F mutation is frequently present at diagnosis in patients with essential thrombocythemia and polycythemia vera Blood 2006 108 1865 1867 10.1182/blood-2006-01-013540 16728702

[B24] Passamonti F Rumi E Pietra D Elena C Boveri E Arcaini L Roncoroni E Astori C Merli M Boggi S A prospective study of 338 patients with polycythemia vera: the impact of JAK2 (V617F) allele burden and leukocytosis on fibrotic or leukemic disease transformation and vascular complications Leukemia 2010 24 1574 1579 10.1038/leu.2010.148 20631743

[B25] Pardanani A Lasho T Finke C Hanson C Tefferi A Prevalence and clinicopathologic correlates of JAK2 exon 12 mutations in JAK2V617F-negative polycythemia vera Leukemia 2007 21 1960 1963 10.1038/sj.leu.2404810 17597810

[B26] Passamonti F Elena C Schnittger S Skoda RC Green AR Girodon F Kiladjian J-J McMullin MF Ruggeri M Besses C Molecular and clinical features of the myeloproliferative neoplasm associated with JAK2 exon 12 mutations Blood 2011 117 2813 2816 10.1182/blood-2010-11-316810 21224469

[B27] Kim JT Cho YG Choi SI Lee YJ Kim HR Jang SJ Moon DS Park YJ Park G JAK2 V617F and Exon 12 Genetic Variations in Korean Patients with BCR/ABL1-negative Myeloproliferative Neoplasms Korean J Lab Med 2010 30 567 574 10.3343/kjlm.2010.30.6.567 21157140

[B28] Ohyashiki JH Hisatomi H Shimizu S Sugaya M Ohyashiki K Detection of low allele burden of JAK2 exon 12 mutations using TA-cloning in patients with erythrocytosis Jpn J Clin Oncol 2009 39 509 513 10.1093/jjco/hyp048 19491085

[B29] Yeh Y-M Chen Y-L Cheng H-Y Su W-C Chow N-H Chen T-Y Ho C-L High percentage of JAK2 exon 12 mutation in Asian patients with polycythemia vera Am J Clin Pathol 2010 134 266 270 10.1309/AJCPK7KGOWPHYWM0 20660330

[B30] Schnittger S Bacher U Haferlach C Geer T Müller P Mittermüller J Petrides P Schlag R Sandner R Selbach J Detection of JAK2 exon 12 mutations in 15 patients with JAK2V617F negative polycythemia vera Haematologica 2009 94 414 418 10.3324/haematol.13223 19252176 PMC2649350

[B31] Rumi E Harutyunyan AS Pietra D Milosevic JD Casetti IC Bellini M Them NC Cavalloni C Ferretti VV Milanesi C CALR exon 9 mutations are somatically acquired events in familial cases of essential thrombocythemia or primary myelofibrosis Blood 2014 123 2416 2419 10.1182/blood-2014-01-550434 24553179

[B32] Lundberg P Karow A Nienhold R Looser R Hao-Shen H Nissen I Girsberger S Lehmann T Passweg J Stern M Clonal evolution and clinical correlates of somatic mutations in myeloproliferative neoplasms Blood 2014 123 2220 2228 10.1182/blood-2013-11-537167 24478400

[B33] Tefferi A Pardanani A Lim K Abdel-Wahab O Lasho T Patel J Gangat N Finke C Schwager S Mullally A TET2 mutations and their clinical correlates in polycythemia vera, essential thrombocythemia and myelofibrosis Leukemia 2009 23 905 911 10.1038/leu.2009.47 19262601 PMC4654629

[B34] Tefferi A Novel mutations and their functional and clinical relevance in myeloproliferative neoplasms: JAK2, MPL, TET2, ASXL1, CBL, IDH and IKZF1 Leukemia 2010 24 1128 1138 10.1038/leu.2010.69 20428194 PMC3035972

[B35] Stegelmann F Bullinger L Schlenk R Paschka P Griesshammer M Blersch C Kuhn S Schauer S Döhner H Döhner K DNMT3A mutations in myeloproliferative neoplasms Leukemia 2011 25 1217 1219 10.1038/leu.2011.77 21537334

[B36] Brecqueville M Rey J Bertucci F Coppin E Finetti P Carbuccia N Cervera N Gelsi‐Boyer V Arnoulet C Gisserot O Mutation analysis of ASXL1, CBL, DNMT3A, IDH1, IDH2, JAK2, MPL, NF1, SF3B1, SUZ12, and TET2 in myeloproliferative neoplasms Genes Chromosomes Cancer 2012 51 743 755 10.1002/gcc.21960 22489043

[B37] Vorechovsky I Jones A Cross N Why do we see JAK2 exon 12 mutations in myeloproliferative neoplasms&quest Leukemia 2013 27 1930 1932 10.1038/leu.2013.85 23511126

[B38] Rotunno G Mannarelli C Guglielmelli P Pacilli A Pancrazzi A Pieri L Fanelli T Bosi A Vannucchi AM Impact of calreticulin mutations on clinical and hematological phenotype and outcome in essential thrombocythemia Blood 2014 123 1552 1555 24371211 10.1182/blood-2013-11-538983

[B39] Rumi E Pietra D Ferretti V Klampfl T Harutyunyan AS Milosevic JD Them NC Berg T Elena C Casetti IC JAK2 or CALR mutation status defines subtypes of essential thrombocythemia with substantially different clinical course and outcomes Blood 2014 123 1544 1551 10.1182/blood-2014-01-550434 24366362 PMC3945864

[B40] Tefferi A Wassie E Lasho T Finke C Belachew A Ketterling R Hanson C Pardanani A Gangat N Wolanskyj A Calreticulin mutations and long-term survival in essential thrombocythemia Leukemia 2014 ᅟ ᅟ [Epub ahead of print] 10.1038/leu.2014.148 24791854

[B41] Gianelli U Iurlo A Cattaneo D Lambertenghi-Deliliers G Cooperation between pathologists and clinicians allows a better diagnosis of Philadelphia chromosome-negative myeloproliferative neoplasms Expert Rev Hematol 2014 7 255 264 10.1586/17474086.2014.876898 24524231

