# Supplementary figures and images for: The mutation profile of JAK2 and CALR in Chinese Han patients with Philadelphia chromosome-negative myeloproliferative neoplasms
Source: J Hematol Oncol. 2014 Jul 15;7:48. doi: 10.1186/s13045-014-0048-6 (PMC4223390; doi:10.1186/s13045-014-0048-6)

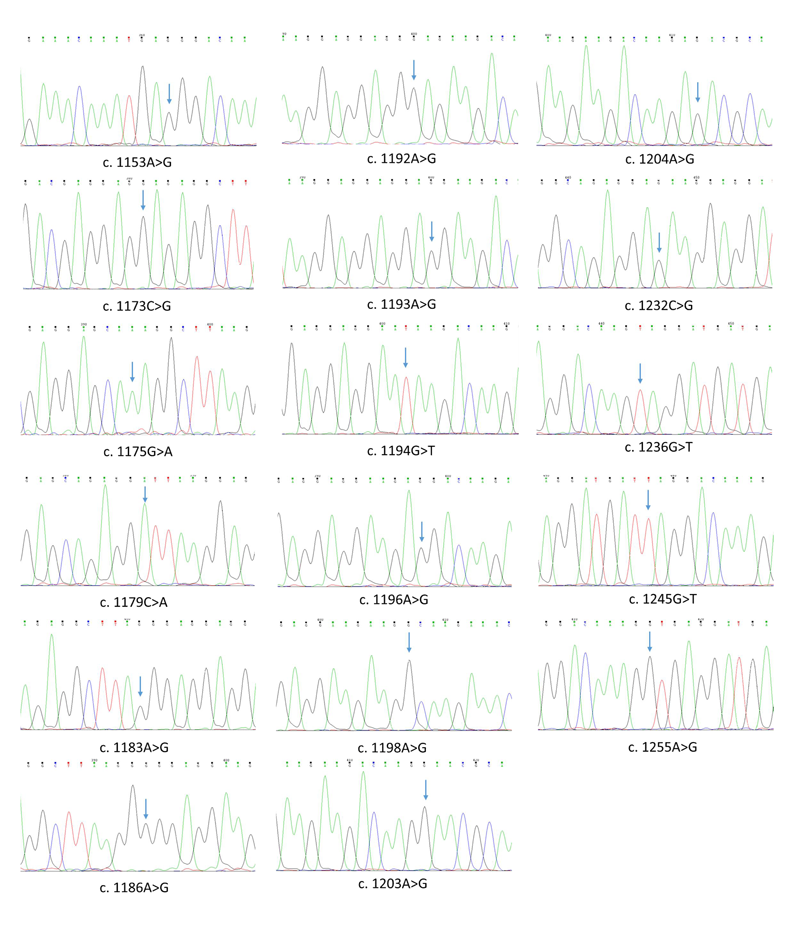

Supplement: Additional file 1: Figure S1. — Seventeen Scattered Point Mutations in CALR Exon 9. [file s13045-014-0048-6-S1.tiff]
